# Carbohydrate co-ingestion with protein does not further augment post-prandial muscle protein accretion in older men

**DOI:** 10.1186/1743-7075-10-15

**Published:** 2013-01-25

**Authors:** Henrike M Hamer, Benjamin T Wall, Alexandra Kiskini, Anneke de Lange, Bart BL Groen, Jaap A Bakker, Annemie P Gijsen, Lex B Verdijk, Luc JC van Loon

**Affiliations:** 1 Department of Human Movement Sciences, Maastricht University Medical Centre, PO Box 616, Maastricht, MD, 6200, The Netherlands; 2 Department of Clinical Genetics, Maastricht University Medical Centre, Maastricht, The Netherlands; 3 Department of Human Biology, Maastricht University Medical Centre, Maastricht, The Netherlands

**Keywords:** Skeletal muscle, Ageing, Sarcopenia, Amino acids, Anabolic resistance

## Abstract

**Background:**

A blunted muscle protein synthetic response to protein ingestion may contribute to the age related loss of muscle tissue. We hypothesized that the greater endogenous insulin release following co-ingestion of carbohydrate facilitates post-prandial muscle protein accretion after ingesting a meal-like bolus of protein in older males.

**Methods:**

Twenty-four healthy older men (75±1 y) were randomly assigned to ingest 20 g intrinsically L-[1-^13^C] phenylalanine-labeled casein protein with (PRO-CHO) or without (PRO) 40 g carbohydrate. Ingestion of specifically produced intrinsically L-[1-^13^C] phenylalanine labeled protein allowed us to assess post-prandial incorporation of dietary protein derived amino acids into muscle protein. Blood samples were collected at regular intervals, with muscle biopsies being obtained prior to and 2 and 6 h after protein ingestion.

**Results:**

Plasma glucose and insulin concentrations showed a greater increase in PRO-CHO compared with PRO (*P*<0.001). Muscle protein-bound L-[1-^13^C] phenylalanine enrichments tended to increase to a greater extent in PRO-CHO compared with PRO during the first 2 h after protein ingestion (0.0072±0.0013 vs 0.0046±0.010 MPE, respectively; *P*=0.13). However, 6 h after protein ingestion, differences in muscle protein-bound L-[1-^13^C] phenylalanine enrichments were no longer observed between experiments (0.0213±0.0024 vs 0.0185±0.0010 MPE, respectively; *P*=0.30).

**Conclusions:**

This study shows that carbohydrate ingestion may accelerate, but does not further augment post-prandial incorporation of dietary protein derived amino acids into muscle protein in healthy elderly men.

## Background

Ageing is associated with the loss of muscle mass and strength, which reduces functional capacity and increases the risk of developing chronic metabolic diseases [1]. Recent work suggests that with ageing skeletal muscle tissue becomes less sensitive to the main anabolic stimuli, i.e. food intake and physical activity [2-5]. The proposed blunted muscle protein synthetic response to protein ingestion, coined “anabolic resistance”, has been suggested to represent a key factor responsible for the progressive loss of skeletal muscle mass with ageing [2-6]. Consequently, many research groups have started to look for dietary strategies that stimulate post-prandial muscle protein synthesis [7-9]. Dietary protein has been identified as the main factor driving the muscle protein synthetic response to food intake [10]. However, other macronutrients may modulate the post-prandial muscle protein synthetic response to protein ingestion.

Previous work suggests that carbohydrate co-ingestion with protein stimulates post-prandial muscle protein accretion [10-14]. The latter could be attributed to the higher energy intake and/or the greater post-prandial insulin release following carbohydrate co-ingestion. In agreement, higher insulin concentrations have been reported to stimulate muscle protein synthesis rates under conditions of hyperaminoacidemia [15]. However, there is an on-going debate on whether circulating insulin levels regulate muscle protein synthesis [16,17]. Based on a series of clamp studies that assessed muscle protein synthesis rates at various levels of circulating insulin [18,19], it has been proposed that insulin is merely permissive, rather than modulatory, to stimulate muscle protein synthesis in healthy young adults [16].

In contrast, this paradigm regarding the proposed permissive role of post-prandial insulin concentrations may not be applicable in older individuals. Higher insulin concentrations may be required to allow an appropriate post-prandial rise in muscle protein synthesis rates in the elderly population. The stimulation of endogenous insulin release following food intake represents a key factor driving post-prandial perfusion, allowing subsequent amino acid delivery to the muscle, facilitating amino acid uptake and/or activating anabolic signalling [20]. The decreased insulin sensitivity observed with ageing is associated with blunted endothelial-dependent vasodilation [21]. It has therefore been proposed that senescent muscle is more resistant to the stimulating effect of post-prandial insulin levels on muscle perfusion and, as such, amino acid delivery to the muscle [22,23]. In agreement, work by Fujita *et al.*[11] has shown that local insulin administration in older adults increases muscle protein synthesis rates. These studies suggest that increasing post-prandial insulin release following food intake may augment amino acid delivery to the muscle, thereby increasing post-prandial muscle protein synthesis rates in the older population.

We hypothesize that co-ingesting carbohydrate increases the post-prandial muscle protein synthetic response to the ingestion of a meal-like bolus of protein in healthy older men. Therefore, we selected 24 older men (age: 75±1 y) in whom we determined the muscle protein synthetic response to the ingestion of 20 g dietary protein with or without 40 g carbohydrate during a 6 h post-prandial period. As presented in previous work from our laboratory [8,24-26] we applied specifically produced intrinsically L-[1-^13^C] phenylalanine labeled casein protein with a high enrichment level (37.4 MPE) to assess post-prandial incorporation of dietary protein derived L-[1-^13^C] phenylalanine into muscle protein.

The present study is the first to provide evidence that greater energy intake or high post-prandial insulin levels are not required to increase the post-prandial muscle protein synthetic response to the ingestion of a meal like bolus of dietary protein ingestion in older individuals.

## Methods

### Subjects

Twenty-four healthy elderly men (age: 75±1 y, body mass index (BMI): 25.8±0.4 kg∙m^-2^) were selected to participate in the present study. Only male subjects were included, since protein synthesis rates have been shown to differ between men and women [27]. The subjects were randomly assigned to an experiment in which a single meal like bolus of protein with (PRO-CHO) or without (PRO) additional carbohydrate was ingested. Subjects’ characteristics are presented in Table 1. Exclusion criteria were BMI >30 kg∙m^-2^, diabetes, all co-morbidities interacting with mobility and muscle metabolism of the lower limbs (e.g. arthrosis, arthritis, spasticity/rigidity, all neurological disorders and paralysis), use of anticoagulants, blood diseases, phenylketonuria, allergy for lidocain and participation in any regular exercise program. This study was conducted according to the guidelines laid down in the Declaration of Helsinki and all procedures involving human subjects were approved by the Medical Ethics Committee of the Maastricht University Medical Centre. Written informed consent was obtained from all subjects.

**Table 1:** Subjects’ characteristics

	**PRO**	**PRO-CHO**
*n*	12	12
Age (y)	74.3 ± 1.2	74.8 ± 1.0
Weight (kg)	78.4 ± 1.9	78.4 ± 2.1
BMI (kg/m^2^)	25.7 ± 0.4	25.8 ± 0.7
Systolic blood pressure (mmHg)	140 ± 5	145 ± 5
Diastolic blood pressure (mmHg)	74 ± 3	71 ± 4
Fat (%)	22.3 ± 1.3	23.2 ± 1.1
Lean body mass (kg)	58.5 ± 1.1	57.5 ± 1.1
Basal plasma glucose (mmol^.^L^-1^)	5.4 ± 0.1	5.4 ± 0.1
Plasma glucose OGTT t=120 min (mmol^.^L^-1^)	6.3 ± 0.5	6.0 ± 0.6
Basal plasma insulin (mU^.^L^-1^)	21 ± 2	21 ± 2
Plasma insulin OGTT t=120 min (mU^.^L^-1^)	93 ± 19	94 ± 16
HbA1c (%)	5.7 ± 0.1	5.7 ± 0.1
HOMA	5.1 ± 0.5	5.1 ± 0.6
OGIS (mL^.^min^.^m^-1^)	347 ± 17	346 ± 16

Values represent means±SEM. HbA_1c_: glycated hemoglobin; OGTT: oral-glucose-tolerance-test; HOMA: Homeostasis Model Assessment; OGIS: Oral Glucose Insulin Sensitivity. Data were analysed with a two-tailed unpaired t-test. No significant differences between groups.

### Pretesting

Prior to selection, all subjects participated in a routine medical screening. Subjects completed a health and activity questionnaire and underwent an oral-glucose-tolerance test (OGTT) to screen for type 2 diabetes according to the criteria set out by the World Health Organization [28]. Venous plasma glucose and insulin concentrations determined during the OGTT were used to determine the oral glucose insulin sensitivity (OGIS) index [29] and the homeostasis model assessment (HOMA) index [30]. Prior to the OGTT, body weight and height were measured, and body composition was determined by dual-energy X-ray absorptiometry (DXA, Discovery A; Hologic, Bedford, USA).

### Diet and activity before testing

All subjects consumed a standardized meal (33±2 kJ^.^kg body weight, providing 44% energy (En%) carbohydrate, 22 En% protein, and 34 En% fat) the evening prior to the experiment. All volunteers refrained from strenuous physical activity and maintained their habitual diet for at least 2 d preceding the experiment.

### Protocol

At 0800, after an overnight fast, subjects arrived at the laboratory by car or public transport. A polytetrafluoroethylene catheter was inserted into a heated dorsal hand vein after which the hand was placed in a hot box (60°C) to allow arterialized venous blood sampling [31]. After a basal arterialized blood sample was collected, a fasting muscle biopsy sample was obtained from the *vastus lateralis* muscle. Subjects then ingested a single bolus of test drink containing 20 g intrinsically L-[1-^13^C] phenylalanine labeled casein. In the PRO-CHO group, 40 g carbohydrate (50% dextrose monohydrate; Avebe Food, Veendam, the Netherlands, 50% maltodextrin; AppliChem GmbH, Darmstand, Germany) was added to the test drink. The consumption of the test drink with or without carbohydrate marked the beginning (*t*= 0 min) of a 6 h post-prandial assessment period. Arterialized blood samples were subsequently collected at *t* = 15, 30, 45, 60, 90, 120, 150, 180, 210, 240, 270, 300, 330, and 360 min. A second muscle biopsy sample was taken from the same limb, through a new incision, >2 cm distal from the first incision at *t* = 120 min. A third muscle biopsy sample was collected from the contralateral leg at *t*= 360 min. Arterialized venous blood samples were collected into pre-chilled EDTA-containing tubes and centrifuged at 1000*g* for 10 min at 4°C. Aliquots of plasma were frozen in liquid nitrogen and stored at −80°C until further analysis. Muscle biopsy samples were obtained from the middle region of the *vastus lateralis*, 15 cm above the patella and ~3 cm below entry through the fascia using the percutaneous needle biopsy technique [32]. Muscle biopsy samples were carefully dissected and freed from any visible non-muscle material and then immediately frozen in liquid nitrogen and stored at −80°C until further analysis.

### Preparation of intrinsically labeled protein

Intrinsically L-[1-^13^C] phenylalanine-labeled micellar casein protein was obtained by infusing a Holstein cow with large quantities of L-[1-^13^C] phenylalanine, collecting milk, and purifying the casein fraction as described previously [33]. The L-[1-^13^C] phenylalanine enrichment in the casein fraction averaged 37.4 mole percent excess (MPE). The casein protein met all chemical and bacteriological specifications for human consumption. Subjects received a total beverage volume of 450 mL, which provided 20 g casein protein. Drinks were flavoured by adding 2 mL vanilla flavour (Givaudan, Naarden, the Netherlands) per L beverage.

### Plasma analyses

Plasma glucose concentrations were analysed with an automatic analyser ABX Pentra 400 (Horiba ABX Diagnostics, Nijmegen, the Netherlands) using an ABX Pentra Glucose HK CP Kit (A11A01667, Horiba ABX Diagnostics). Insulin was analysed by radioimmunoassay using a commercially available kit (Insulin RIA kit HI-14K; Millipore, Billerica, USA). For determination of plasma amino acid concentrations, 10 μl of plasma was mixed with 1500 μl of 0.5 mM Tridecafluoroheptanoic acid (TDFHA) (Sigma, Zwijndrecht, The Netherlands) in water and 10 μl of the internal standard solution containing stable isotope-labeled amino acids (Cambridge Isotope Laboratories, Inc., Andover, USA) in 0.1 M HCl [34]. Amino acid concentrations were determined using ultra-performance liquid chromatography tandem mass spectrometry (UPLC-MS/MS) as described previously [34].

For plasma phenylalanine enrichment measurements, plasma phenylalanine was derivatised to its *t*-butyldimethylsilyl (TBDMS) derivative, and the [1-^13^C] phenylalanine enrichment was determined by electron ionization gas chromatography–mass spectrometry (GC-MS, model 7890AN GC/5975C MSD; Agilent, Little Falls, USA) by using selected ion monitoring of masses 336 and 337 for unlabeled and 1-^13^C labeled phenylalanine, respectively [35]. Standard regression curves were applied in all isotopic enrichment analyses to assess the linearity of the mass spectrometer and to control for the loss of tracer. Enrichments (MPE) were corrected for the natural abundance of ^13^C phenylalanine [36].

### Muscle tissue analyses

For the measurement of L-[1-^13^C] phenylalanine enrichment in the tissue-free amino acid pool and mixed muscle protein, 40–60 mg wet muscle was freeze dried. Collagen, blood, and other non-muscle fibre material were removed from the muscle fibres under a light microscope. The isolated muscle fibre mass was weighed, and 35 volumes (7 × dry weight of isolated muscle fibres × wet/dry weight ratio) of ice-cold 2% perchloric acid were added. The tissue was then homogenized by sonification (3 × 10 sec with 10 sec in-between time intervals) and centrifuged (at 1160 *g* for 20 min at 4°C). The supernatant was collected and processed in the same manner as the plasma samples, such that tissue-free L-[1-^13^C] phenylalanine enrichment could be measured by using its TBDMS derivative on a GC-MS. The protein pellet was washed with 3 additional 1 mL washes of 2% perchloric acid, dried, and hydrolysed in 6 M HCl at 120°C for 15 to 18 h. The hydrolysed protein fraction was dried under a nitrogen stream while heated to 120°C. A 50% acetic acid solution was then added, and the hydrolysed protein was passed over a Dowex exchange resin (AG 50W-X8, 100–200 mesh hydrogen form; Bio-Rad, Hercules, USA) by using 1M HCl, H_2_O and 2 M NH_4_OH as eluents. The NH_4_OH fraction was collected and used for further analysis. L-[1-^13^C] phenylalanine enrichment was determined by derivatisation to its *N(O,S)*-ethoxycarbonyl ethyl ester. The enrichment of the derivative was measured by GC-C-IRMS (MAT 253; Thermo Scientific, Bremen, Germany) by using an HP-Ultra 1 GC-column (no. 19091A-112; Hewlett Packard, USA), GC Isolink, and monitoring of ion masses 44, 45 and 46. By establishing the relationship between the enrichment of a series of L-[1-^13^C] phenylalanine standards of variable enrichment and the enrichment of the *N(O,S)*-ethoxycarbonyl ethyl esters of these standards, the muscle protein-bound enrichment of phenylalanine was determined. Standard regression curves were applied to assess the linearity of the mass spectrometer and to control for the loss of tracer. The coefficient of variation for the measurement of L-[1-^13^C] phenylalanine enrichment in mixed muscle protein averaged 0.46±0.02%.

### Calculation of mixed muscle protein fractional synthetic rate from the ingested casein

The muscle protein fractional synthetic rate (FSR) from the ingested casein over the 6 h post-prandial period was calculated using the standard precursor product relationship [37]. Muscle FSR was calculated as follows:

(1) $$\mathrm{FSR}=\frac{\mathrm{\Delta Ep}}{\mathrm{Eprecursor}\times t}\times 100$$

Where *ΔE*p is the delta increment of muscle protein bound L-[1-^13^C] phenylalanine enrichment (MPE) during the incorporation period, *E*precursor is the average values of plasma L-[1-^13^C] phenylalanine or muscle free enrichments (MPE) during the incorporation period and *t* is the time interval (h) between biopsy samples [38].

### Statistics

All data are expressed as means±SEMs. The plasma insulin and glucose responses were calculated as the positive incremental area under the curve (iAUC) above baseline values. Differences in baseline values (i.e. age, weight, BMI, body composition, blood pressure, glucose tolerance) were determined using an unpaired, two-tailed Student’s t-Test. Two-way ANOVA with time as within subjects factor and group (i.e. treatment) as between subjects factor was used to compare differences between treatments over time in plasma glucose and insulin concentrations, plasma amino acid concentrations and plasma L-[1-^13^C] phenylalanine enrichments. In case of a significant interaction between time and treatment, Bonferroni post-tests were applied to locate the differences. Differences between treatments in iAUC of plasma glucose and insulin, muscle free and protein-bound L-[1-^13^C] phenylalanine enrichments and mixed muscle FSR were analysed with an unpaired, two-tailed Student’s t-Test. Correlations between plasma and muscle free L-[1-^13^C] phenylalanine enrichments were assessed by calculation Pearson’s correlations coefficients. Statistical significance was set at P<0.05. All calculations were performed by using the PASW statistics 18.0.3 software package.

## Results

### Plasma glucose and insulin

**Figure 1:**
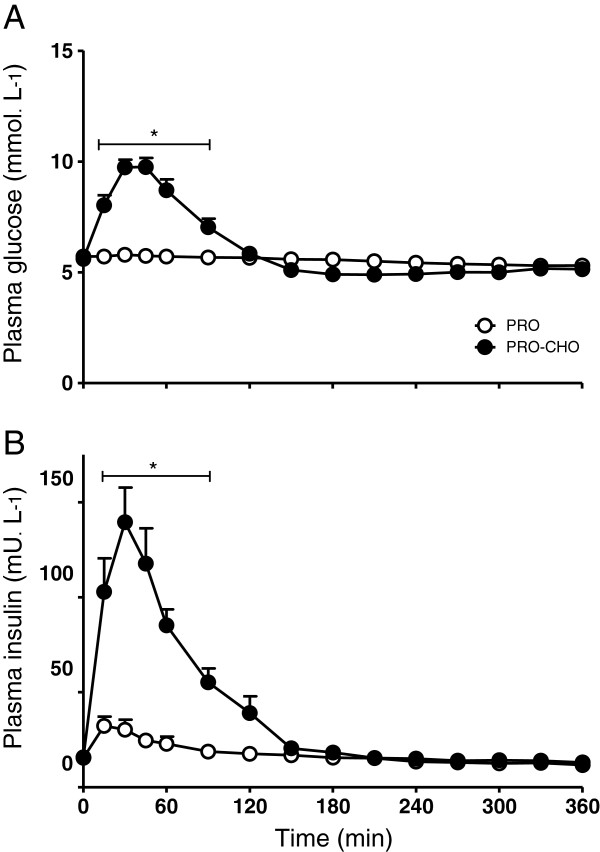
**plasma glucose (A) and insulin (B) concentrations.** Mean (±SEM) plasma glucose (**A**) and insulin (**B**) concentrations following ingestion of 20 g casein with (PRO-CHO; n=12) or without (PRO; n=12) 40 g carbohydrate in healthy older men. Glucose: significant time (P<0.0001), treatment (P<0.0001) and interaction (P<0.0001) effect. Insulin: significant time (P<0.0001), treatment (P=0.0002) and interaction (P<0.0001) effect. * = significantly greater when compared with the PRO experiment (P<0.05).

Plasma glucose and insulin concentrations in the PRO and PRO-CHO experiment are shown in Figure 1. Plasma glucose concentrations (Figure 1A) significantly increased after ingestion of casein plus carbohydrate (PRO-CHO), whereas no significant increase was observed after ingestion of casein only (Significant time (P<0.0001), treatment (P<0.0001) and interaction (P<0.0001) effect). In accordance, the plasma glucose response (iAUC) was significantly greater in the PRO-CHO compared with the PRO experiment (288±34 vs. 13±5 mmol^.^L^-1.^6 h^-1^, respectively; P<0.0001). Plasma insulin concentrations (Figure 1B) showed a rapid increase following drink ingestion in both groups, although plasma insulin concentrations reached significantly higher concentrations in the PRO-CHO compared with the PRO experiment (Significant time (P<0.0001), treatment (P=0.0002) and interaction (P<0.0001) effect). In accordance, the plasma insulin response (iAUC) was significantly greater in the PRO-CHO compared with the PRO experiment (8421±1054 vs. 1058±234 mU^.^L^-1.^6 h^-1^, respectively; P<0.0001). Plasma amino acids

**Figure 2:**
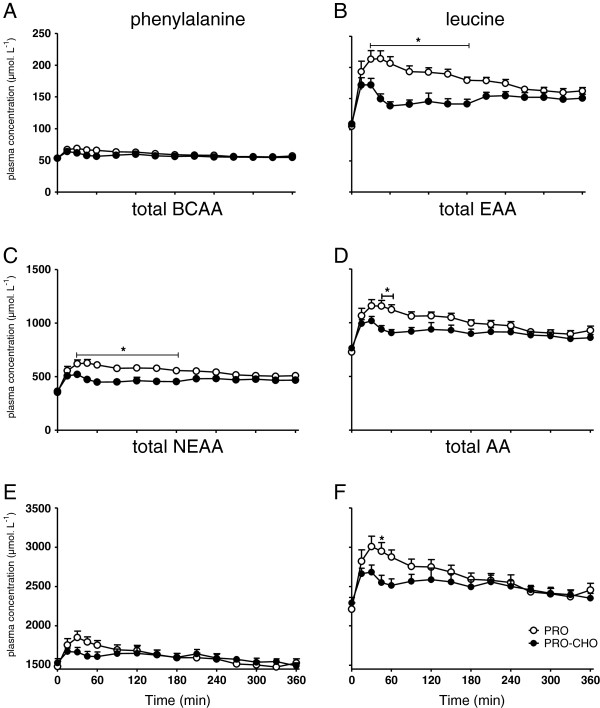
**Mean (±SEM) plasma phenylalanine (A), leucine (B), total branched chain amino acids (BCAA; C), total essential amino acids (EAA; D), total non-essential amino acids (NEAA; E) and total amino acid (AA; F) concentrations following ingestion of 20 g casein with (PRO-CHO; n=12) or without (PRO; n=12) 40 g carbohydrate in healthy older men.** Phenylalanine: significant time (P<0.0001) and interaction (P=0.0441) effect. Leucine: significant time (P<0.0001), treatment (P<0.0001) and interaction (P<0.0001) effect. Total BCAA: significant time (P<0.0001), treatment (P=0.0006) and interaction (P<0.0001) effect. Total EAA: significant time (P<0.0001), treatment (P=0.0182) and interaction (P=0.0028) effect. Total NEAA: significant time (P<0.0001) and interaction (P=0.0004) effect. Total AA: significant time (P<0.0001) and interaction (P<0.0012) effect. *= significantly lower when compared with the PRO experiment (P<0.05).

Plasma phenylalanine (A), leucine (B), total essential amino acid (EAA; C), total branched chain amino acid (BCAA; D), total amino acid (E) and non-essential amino acid (NEAA; F) concentrations over time are depicted in Figure 2. Following protein ingestion, a rapid increase in plasma amino acid concentrations was observed in both experiments (P<0.0001). Plasma phenylalanine concentrations did not differ between experiments, whereas plasma leucine concentrations were significantly lower in the PRO-CHO compared with the PRO experiment between 30–180 min following ingestion of the drink. In line, total plasma BCAA concentrations were significantly lower in the PRO-CHO compared with the PRO experiment group during the same time period. Total plasma EAA concentrations were significantly lower in the PRO-CHO experiment between 45–60 min following protein ingestion (P<0.05). Total plasma NEAA concentrations did not differ significantly between experiments. Plasma L-[1-^13^C] phenylalanine enrichments

**Figure 3:**
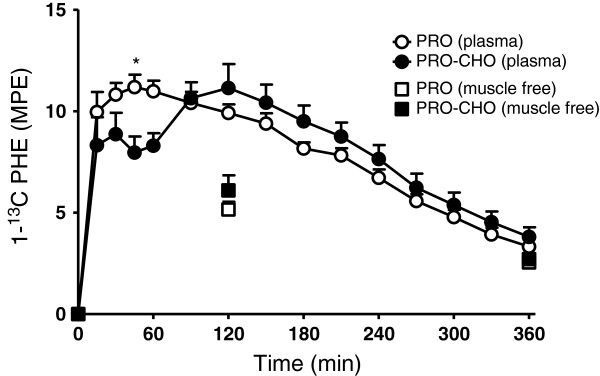
**Mean (±SEM) plasma and muscle free L-[1-**^**13**^**C] phenylalanine enrichments following ingestion of 20 g intrinsically L-[1-**^**13**^**C] phenylalanine-labeled casein protein with (PRO-CHO; n=12) or without (PRO; n=12) 40 g carbohydrate in healthy older men.** No significant differences in muscle free L-[1-^13^C] phenylalanine enrichments were observed between experiments. A significant time (P<0.0001) and interaction (P<0.0009) effect was found for plasma L-[1-^13^C] phenylalanine enrichments. *= significantly lower when compared with the PRO experiment (P<0.05).

The increase in plasma L-[1-^13^C] phenylalanine enrichment over time is illustrated in Figure 3. Following ingestion of 20 g intrinsically L-[1-^13^C] phenylalanine-labeled casein, plasma L-[1-^13^C] phenylalanine enrichments increased rapidly in both experiments (significant time (P<0.0001) and interaction (P<0.0009) effect). Peak plasma L-[1-^13^C] phenylalanine enrichments did not differ between groups and averaged 11.2±0.6 and 11.2±1.2 MPE in the PRO and PRO-CHO experiment, respectively. However, peak levels were reached much earlier in the PRO group (at t=45 versus t=120 min, respectively). Muscle tracer analysis

**Figure 4:**
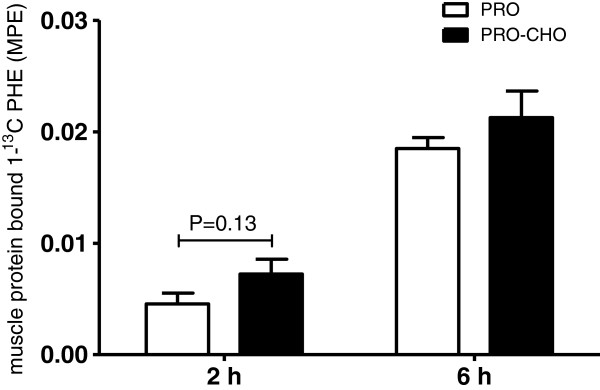
**Mean (±SEM) muscle protein-bound L-[1-**^**13**^**C] phenylalanine enrichments (MPE) at 2 and 6 h following the ingestion of 20 g intrinsically L-[1-**^**13**^**C] phenylalanine-labeled casein with (PRO-CHO; n=12) or without (PRO; n=12) 40 g carbohydrate in healthy older men.** No significant differences were observed between experiments. There was a tendency for greater muscle protein accretion in the CHO+PRO experiment during the early post-prandial period (P=0.13).

Muscle free L-[1-^13^C] phenylalanine enrichments (MPE) at 2 and 6 h following into the post-prandial period are shown in Figure 3. Muscle free L-[1-^13^C] phenylalanine enrichments at 2 h following the drink averaged 5.1±0.4 and 6.1±0.7 MPE in the PRO and PRO-CHO experiment, respectively (P=0.29). At 6 h following the drink muscle free L-[1-^13^C] phenylalanine enrichments had declined to 2.5±0.2 and 2.7±0.3 MPE, respectively (P=0.65). A strong significant correlation was observed between plasma and muscle free L-[1-^13^C] phenylalanine enrichment at 2 h (r=0.89; P<0.0001) and 6 h (r=0.92; P<0.0001). Muscle protein-bound L-[1-^13^C] phenylalanine enrichments (MPE) assessed 2 and 6 h after protein ingestion are shown in Figure 4. Muscle protein-bound L-[1-^13^C] phenylalanine enrichments at 2 h tended to be higher in the PRO-CHO compared with PRO experiment (0.0072±0.0013 vs 0.0046±0.0010 MPE: P=0.13). At 6 h after protein ingestion muscle protein-bound L-[1-^13^C] phenylalanine enrichments no longer differed between experiments and averaged 0.0213±0.0024 and 0.0185±0.0010 MPE in the PRO-CHO and PRO experiment, respectively (P=0.30). Post-prandial amino acid deposition from dietary protein in the muscle protein pool was 59% and 15% greater following carbohydrate co-ingestion (PRO-CHO) when compared to the ingestion of protein only (PRO), at 2 and 6 h after protein ingestion, respectively. Mixed muscle protein fractional synthetic rates calculated over the entire 6 h post-prandial period, with the plasma tracer enrichments used as the precursor pool, did not differ between groups (0.0438±0.0046 vs 0.0400±0.0026 %^.^h^-1^, in the PRO-CHO and the PRO group, respectively; P=0.48). Similarly, when muscle free L-[1-^13^C] phenylalanine enrichments were used as a precursor to calculate FSR over the entire 6 h post-prandial period no differences were observed between groups (0.088±0.009 vs 0.099±0.008, in the PRO-CHO and the PRO group, respectively; P=0.44).

## Discussion

In the present study, older men ingested intrinsically labeled dietary protein to assess the subsequent incorporation of the dietary protein derived amino acids into mixed muscle protein. We compared the post-prandial muscle protein synthetic response after ingesting a single meal-like (20 g) amount of dietary protein with or without 40 g carbohydrate in healthy older men. Carbohydrate co-ingestion strongly stimulated post-prandial insulin release. However, carbohydrate co-ingestion did not augment the post-prandial use of the ingested protein derived amino acids for muscle protein synthesis during a 6 h post-prandial period in healthy older men.

Previous work suggests that co-ingestion of other macronutrients with protein may modulate the post-prandial muscle protein synthetic response [11-15,39,40]. In the present study, we assessed whether post-prandial muscle protein accretion following the ingestion of a meal-like amount of casein protein could be augmented by adding 40 g carbohydrate. Therefore, we applied specifically produced intrinsically L-[1-^13^C] phenylalanine labeled dietary protein which allowed us to assess the metabolic fate of the ingested protein towards its incorporation into muscle protein [33,41]. Importantly, this allows us to investigate the impact of carbohydrate co-ingestion in a more physiological (i.e. bolus feeding) setting. Carbohydrate co-ingestion strongly elevated plasma insulin concentrations with peak values of 138±18 vs. 34±5 mU ^.^ L^-1^, in the PRO-CHO and PRO experiment, respectively. After 90 min, plasma insulin concentrations no longer differed between experimental conditions (Figure 1). In agreement with previous work [42,43], the greater insulin response seemed to modulate the plasma amino acid kinetics (Figure 2). Carbohydrate co-ingestion attenuated the post-prandial rise in plasma amino acid concentrations, mainly the branched chain amino acids (Figure 2). Plasma [1-^13^C] phenylalanine enrichments were also significantly lower in the PRO-CHO vs. PRO experiment, during the first 90 min after protein ingestion (Figure 3). The attenuated rise in plasma phenylalanine concentration and [1-^13^C] phenylalanine enrichment following carbohydrate co-ingestion coincided with the greater rise in circulating insulin levels, implying that (muscle) tissue amino acid uptake was accelerated during the early post-prandial phase in the PRO-CHO experiment.

An insulin induced stimulation of amino acid uptake could allow a more rapid provision of amino acids available for muscle protein synthesis. In accordance, we observed a tendency for a greater incorporation rate of dietary protein derived L-[1-^13^C] phenylalanine into skeletal muscle protein during the first 2 hours after protein ingestion when carbohydrate was co-ingested (P=0.13; Figure 4).

The suggested impact of carbohydrate ingestion on muscle protein accretion during the early post-prandial phase was not maintained. Incorporation of dietary protein derived L-[1-^13^C] phenylalanine into skeletal muscle protein during the 6 h following protein ingestion did not differ between experimental conditions and averaged 0.0213±0.0024 and 0.0185±0.0010 MPE in PRO-CHO and PRO, respectively (P=0.30; Figure 4). From these data we can conclude that carbohydrate co-ingestion may accelerate, but does not modify the metabolic fate of dietary protein derived amino acids for muscle protein synthesis over a 6 h post-prandial period in older men.

Our results suggest that the post-prandial insulin response following ingestion of 20 g casein only was already sufficient to maximize insulin-mediated stimulation of muscle protein synthesis. This finding is consistent with Greenhaff *et al.*[18] who demonstrated that under clamp conditions insulin concentrations of merely 5–15 mU^.^L^-1^ are already sufficient to allow maximal stimulation of muscle protein synthesis rate during hyperaminoacidemic conditions in healthy young men. However, the latter has been questioned for the older population, as insulin resistance in senescent muscle has been associated with impaired insulin stimulated endothelial-dependent vasodilation [13-15]. Therefore, it can be hypothesized that higher post-prandial insulin concentrations are required to maximize the muscle protein synthetic response to food intake in the older population. Though the present data indicate that a greater post-prandial insulin response in the older population may accelerate the use of dietary protein derived amino acids for muscle protein synthesis, this does not modulate the total 6 h post-prandial muscle protein synthetic response. Discrepancies between the present results and previously published work [11-15,39] may be attributed to differences in study design with the other studies using a continuous supply of extra amino acids and/or carbohydrate rather than a single bolus. Nevertheless, this does not mean that the post-prandial muscle protein synthetic response cannot be further increased in the elderly population. Recently, we showed that by ingesting more protein (35 over 20 g), post-prandial dietary protein derived amino acid incorporation rates can be further improved [25]. Though we can only speculate on the impact of carbohydrate co-ingestion and/or greater insulin release on post-prandial muscle protein synthesis following ingestion of greater amounts of dietary protein, these combined findings may be used to suggest that more protein dense meals should be consumed by elderly and/or more clinically compromised populations.

The observation that additional carbohydrate ingestion is not required to optimize post-prandial muscle protein synthesis is of important clinical relevance. With a proposed blunted muscle protein synthetic response in the elderly population, the need for proper dietary intervention is well recognized. Therefore, protein supplementation is presently being used in clinical practice to prevent or attenuate muscle loss in the older population. However, in the light of preventing excess energy intake and hyperglycemic blood glucose excursions it has been questioned what food products or specifically designed nutritional supplements would be preferred to maximize post-prandial muscle protein accretion. The present study shows that large amounts of carbohydrate are definitely not required to maximize post-prandial muscle protein accretion to the ingestion of a single 20 g bolus of dietary protein, suggesting that products high in protein and low in carbohydrate are as effective to stimulate muscle protein synthesis without increasing energy intake.

We conclude that carbohydrate ingestion may accelerate, but does not further augment post-prandial incorporation of dietary protein derived amino acids into muscle protein in healthy elderly men. This implies that ingestion of carbohydrate is not required to optimize post-prandial muscle protein accretion in the older population. This has important clinical relevance for the development of clinical nutrition for the elderly population.

## Abbreviations

BMI: Body mass index; FSR: Fractional synthetic rate; HOMA: Homeostasis model assessment; MPE: Mole percent excess; OGIS: Oral glucose insulin sensitivity; OGTT: Oral-glucose-tolerance test.

## Competing interests

All authors report that there are no financial or non-financial conflicts of interests.

## Authors’ contributions

LBV and LJCvL conceived of the study; HMH, BTW, AK, BBLG and AdL carried out the human intervention study; HMH and BTW performed the statistical analysis; JAB and APG performed the biological analyses; HMH and LJCvL drafted the manuscript. All authors have read and approved the final manuscript.
